# Population history and ecology, in addition to climate, influence human stature and body proportions

**DOI:** 10.1038/s41598-020-79501-w

**Published:** 2021-01-11

**Authors:** Emma Pomeroy, Jay T. Stock, Jonathan C. K. Wells

**Affiliations:** 1 Department of Archaeology, University of Cambridge, Downing Street, Cambridge, CB2 3DZ UK; 2 Department of Anthropology, University of Western Ontario, London, ON N6A 5C2 Canada; 3 UCL Great Ormond Street Institute of Child Health, University College London, Great Ormond Street, London, WC1N 3JH UK

**Keywords:** Evolution, Anthropology

## Abstract

Worldwide variation in human stature and limb proportions is widely accepted to reflect thermal adaptation, but the contribution of population history to this variation is unknown. Furthermore, stature and relative lower limb length (LLL) show substantial plastic responses to environmental stressors, e.g., nutrition, pathogen load, which covary with climate. Thus ecogeographic patterns may go beyond temperature-based selection. We analysed global variation in stature, sitting height and absolute and relative LLL using large worldwide samples of published anthropometric data from adult male (n = 571) and female (n = 268) populations in relation to temperature, humidity, and net primary productivity (NPP). Population history was modeled using spatial eigenvector mapping based on geographic distances reflecting the hypothesized pattern for the spread of modern humans out of Africa. Regression models account for ~ 50% of variation in most morphological variables. Population history explains slightly more variation in stature, sitting height and LLL than the environmental/climatic variables. After adjusting for population history, associations between (usually maximum) temperature and LLL are consistent with Allen's "rule" and may drive similar relationships with stature. NPP is a consistent negative predictor of anthropometry, which may reflect the growth-limiting effects of lower environmental resource accessibility (inversely related to NPP) and/or pathogen load.

## Introduction

In common with other mammals, ecogeographic variation in recent human morphology appears to follow Bergmann’s^1^ and Allen’s “rules”^2^, as with increasing distance from the equator or decreasing ambient temperature, body mass increases^3–7^ and limbs are shorter relative to the trunk^6–9^. Stature^10^, body breadth^11,12^, the relative length of distal limb segments (forearm/lower leg and hands/feet)^13–15^, nasal shape^16–18^ and cranial vault shape^19–23^ and body surface area^24^ also demonstrate clinal variation with latitude. Greater body mass, relatively shorter limbs, a wider body, proportionally shorter distal limb segments and a more rounded cranial vault reduce heat loss in cold environments by decreasing the surface area to volume ratio^5,25^, and suggest the impact of natural selection on human body form. However, other climatic factors such as rainfall and humidity might place differing selective pressures on morphology^26,27^. Unlike in hot dry environments where sweating is an effective thermoregulatory strategy and longer limbs provide greater surface area for evaporative heat loss [although see^28^], in humid conditions evaporative heat loss is ineffective, and reduced metabolic heat production through smaller body mass may be the primary mechanism for limiting heat stress^26^. The potential influence of these different climatic components on body proportions have not been widely investigated [^9^ is a notable exception], and temperature often assumed to be the main driver of ecogeographic patterns, with latitude frequently used as an imperfect proxy in analyses [e.g.,^4–6,29^].

The extent to which phenotype reflects climate per se, rather than other variables that covary with climate which may actually drive ecogeographic variation in body size and proportions, has not been widely explored. Temperature, rainfall, seasonality and humidity influence environmental productivity and thus may affect diet and nutrition^15^. As shorter stature and proportionally shorter limbs are also known to result from poor health and nutrition or other stress during development^30–33^, we might expect greater environmental productivity (reflected by net primary productivity or NPP) to be associated with taller stature and absolutely and relatively longer lower limbs.

However, plants in equatorial areas have more chemical or physical defences that make them less available for animal consumption, have a lower seasonal peak in resource abundance^34–37^ and grow in less fertile soils^35,36^. “Ecologically and evolutionarily relevant NPP” [“eNPP”:^35,36^], essentially NPP during the growing season, peaks around 60 degrees north and south for terrestrial and marine ecosystems, and is thought to drive parallel geographical peaks in body size for a range of mammals including humans^34–37^. NPP (or eNPP) is an imperfect proxy for nutritional adequacy^35^, which for humans is affected by a range of factors including (but not limited to) the availability of specific micronutrients, local variability in ecology, cultural influences on diet and subsistence or the use of aquatic resources in the diet, it serves as a useful proxy for global-scale variation in general resource availability. Alternatively, pathogen load is greater in tropical regions^38^ and has been proposed to lead to greater investment in immune function in both humans^39, 40^ and other animals [e.g.,^41, 42^] at the expense of growth compared with temperate regions.

Furthermore, several recent studies demonstrate that population history and neutral evolutionary processes explain a significant proportion of geographic variation in cranial and pelvic morphology^8,21, 22,43–50^ and body surface area to volume ratio [e.g.,^24^], often more than environmental variables, while also supporting climatic influences on some characteristics^16,20,21,48^. Conversely, results for the signatures of population history have been mixed for limb bone size and proportions^29,46,51^. However, previous investigations of worldwide variation in limb and trunk proportions in living populations have not taken shared ancestry into account, which may confound apparent relationships between morphology and climate.

Consequently, the widely-cited associations between human phenotypic variation and climate still need to be subjected to more nuanced analyses to establish their potential relationships to specific climatic variables, as well as the likely influence of climate-related variables such as environmental productivity, and population history. In this study, we use anthropometry from a large worldwide sample of populations to investigate the association of variation in stature, sitting height, and lower limb length (LLL: both absolute and relative to trunk length) with specific climatic (temperature, humidity) and environmental (NPP) variables, taking into account population history.

## Results

**Figure 1:**
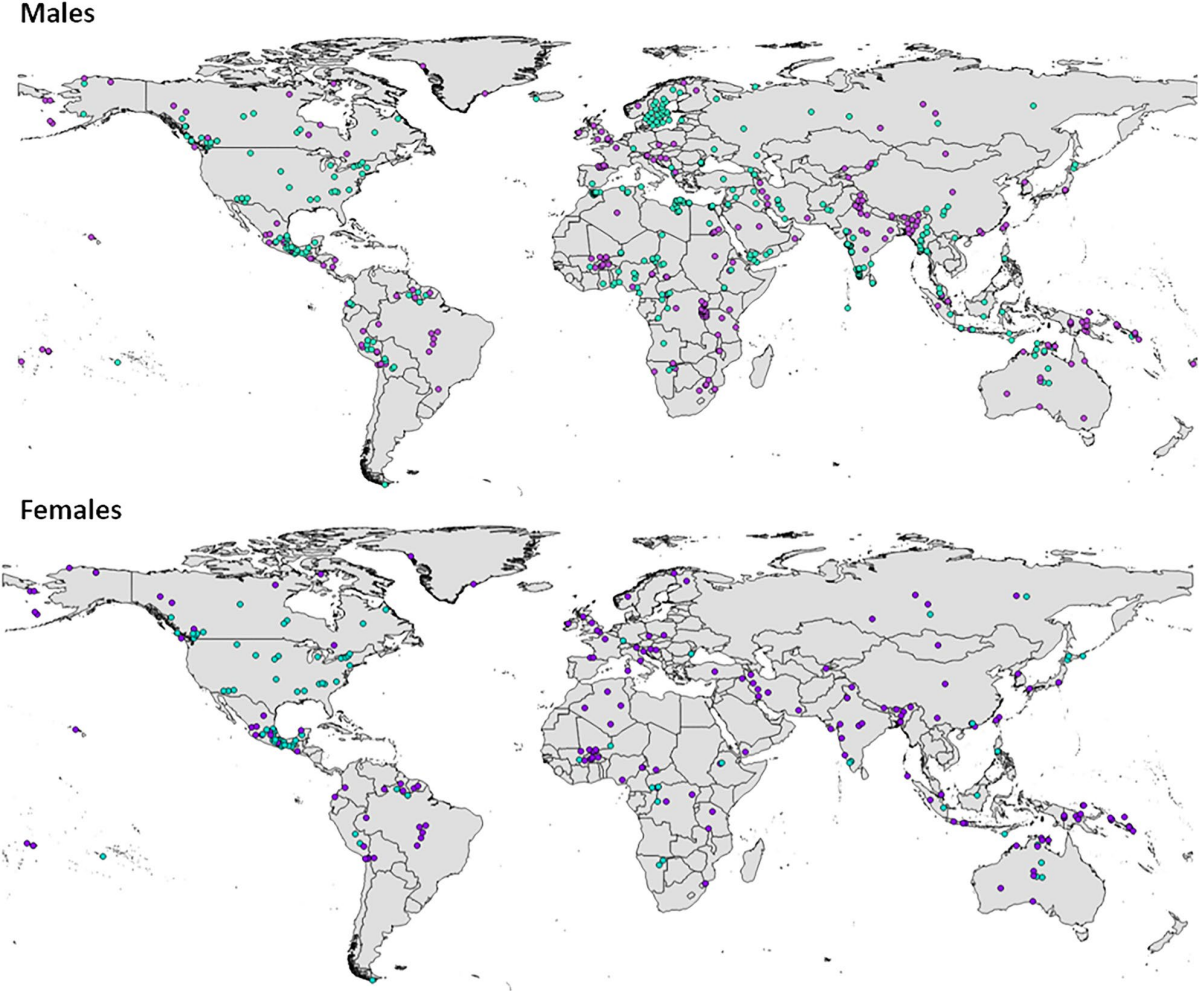
World map showing male and female samples included in this analysis: green = pre-1950, purple = 1950 or later.

**Figure 2:**
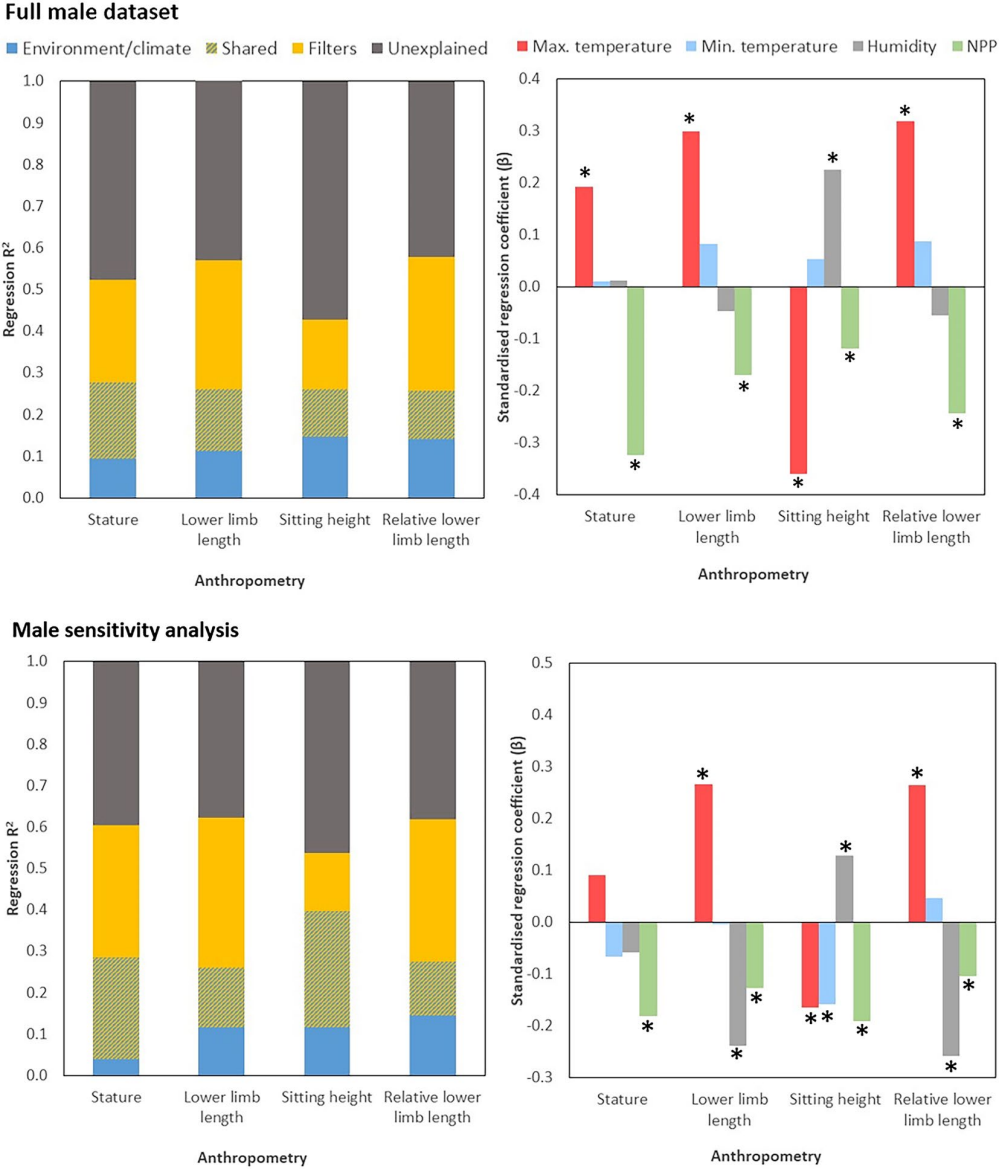
Results from the analysis of relationships between anthropometry and climatic/environmental variables for the male dataset (above: full; below; sensitivity analysis), adjusted for population history using spatial filters. Left: Variation explained by environment/climate, spatial filters, both together, or remaining unexplained by the models. Right: Standardised regression coefficients for environmental/climatic variables in explaining anthropometry, adjusting for population history. *NPP* net primary productivity. * Denotes p < 0.05 for variable in regression model, of which full details are in Suppplementary Tables S5–S6. Above: full sample. Below: sensitivity analysis.

**Figure 3:**
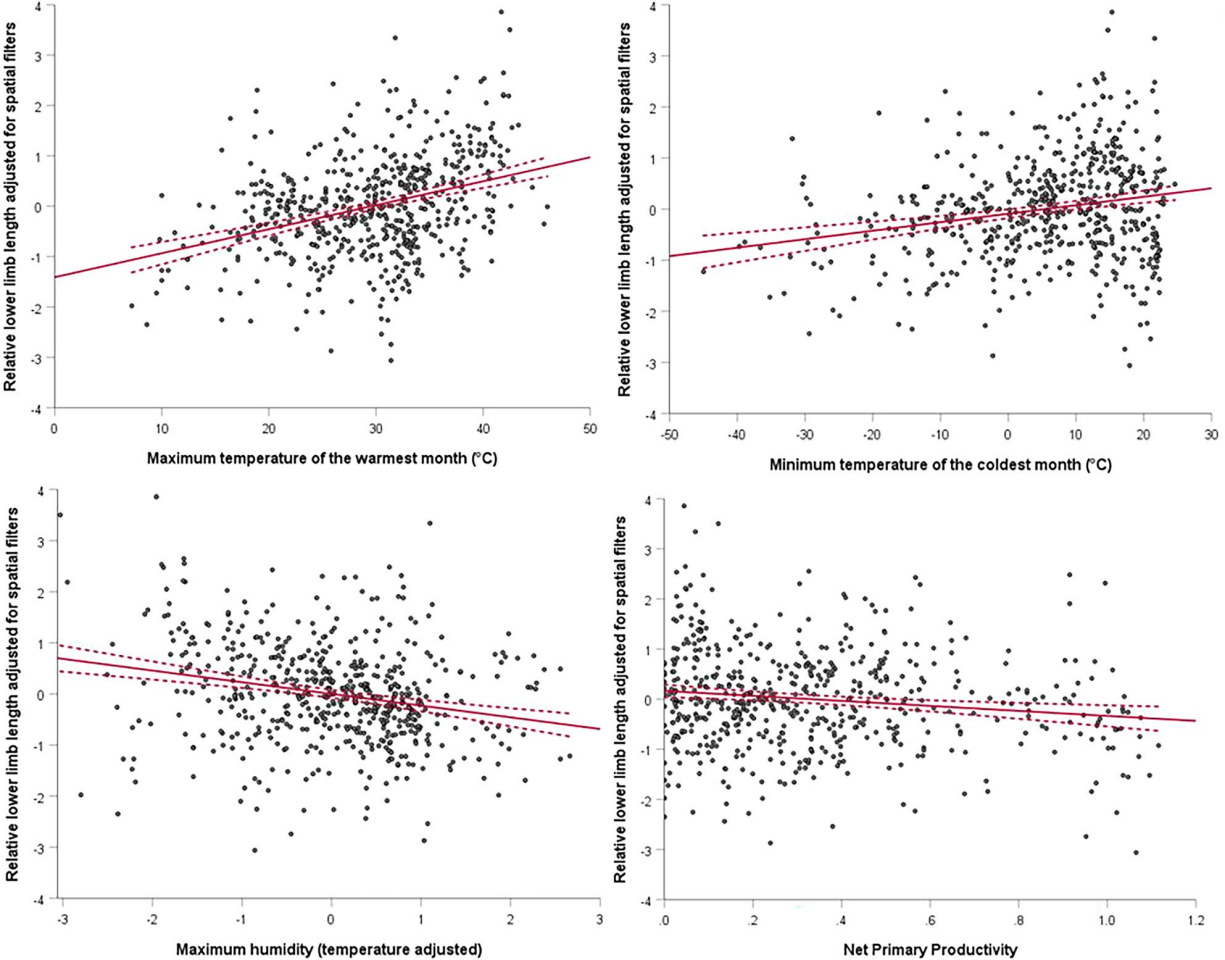
Example scatterplots for male relative lower limb length adjusted for relevant spatial filters, against climatic and environmental variables. Linear regression lines (solid) and 95% confidence intervals (dashed) are shown. Net primary productivity in tonnes of carbon m^−2^ year^−1^ × 10^–6^.

The locations of the study populations are shown in Fig. 1. The full datasets, summary statistics and full analyses are presented in the online supplementary information (Tables S1–S8). For the full male sample (n = 571), regression models explain approximately half the variation in anthropometric outcomes with adjusted R^2^ ranging from 0.46 for sitting height to -0.49 for absolute and relative LLLs. The spatial filters (population history) explain a greater unique proportion of the variance than the environmental/climatic variables, and generally a smaller proportion of the variance is shared by the spatial filters and environmental/climatic variables (Fig. 2). Maximum temperature is positively associated with all anthropometric outcomes except sitting height, while minimum temperature is negatively associated with sitting height and positively associated with LLL (absolute and relative). Humidity is positively associated with sitting height and negatively with absolute and relative LLLs. NPP is negatively associated with all anthropometric outcomes (Figs. 2, 3). The standardised coefficients for temperature and NPP are broadly similar across anthropometric variables, but it is notable that for stature and sitting height, the absolute standardised coefficient is greater for NPP than for temperature, while the reverse is true for LLL (relative and absolute).

The results from the male sensitivity analysis (Fig. 2, Table S6) broadly reflect those from the full sample. It is notable that the proportion of variance explained is slightly higher for the sensitivity analysis (adjusted R^2^ = 0.53–0.61). The spatial filters uniquely explain a greater proportion of the variance than the environmental/climatic variables, and the proportion of variance shared across the spatial filters and environmental/climatic variables is greater than in the previous analysis. Maximum temperature is a significant positive predictor of LLL (relative and absolute), but a negative predictor of sitting height, while minimum temperature is a significant negative predictor of sitting height. Humidity is a negative predictor of lower limb length (relative and absolute) and a positive predictor of sitting height. NPP is again a significant negative predictor of all anthropometry.

**Figure 4:**
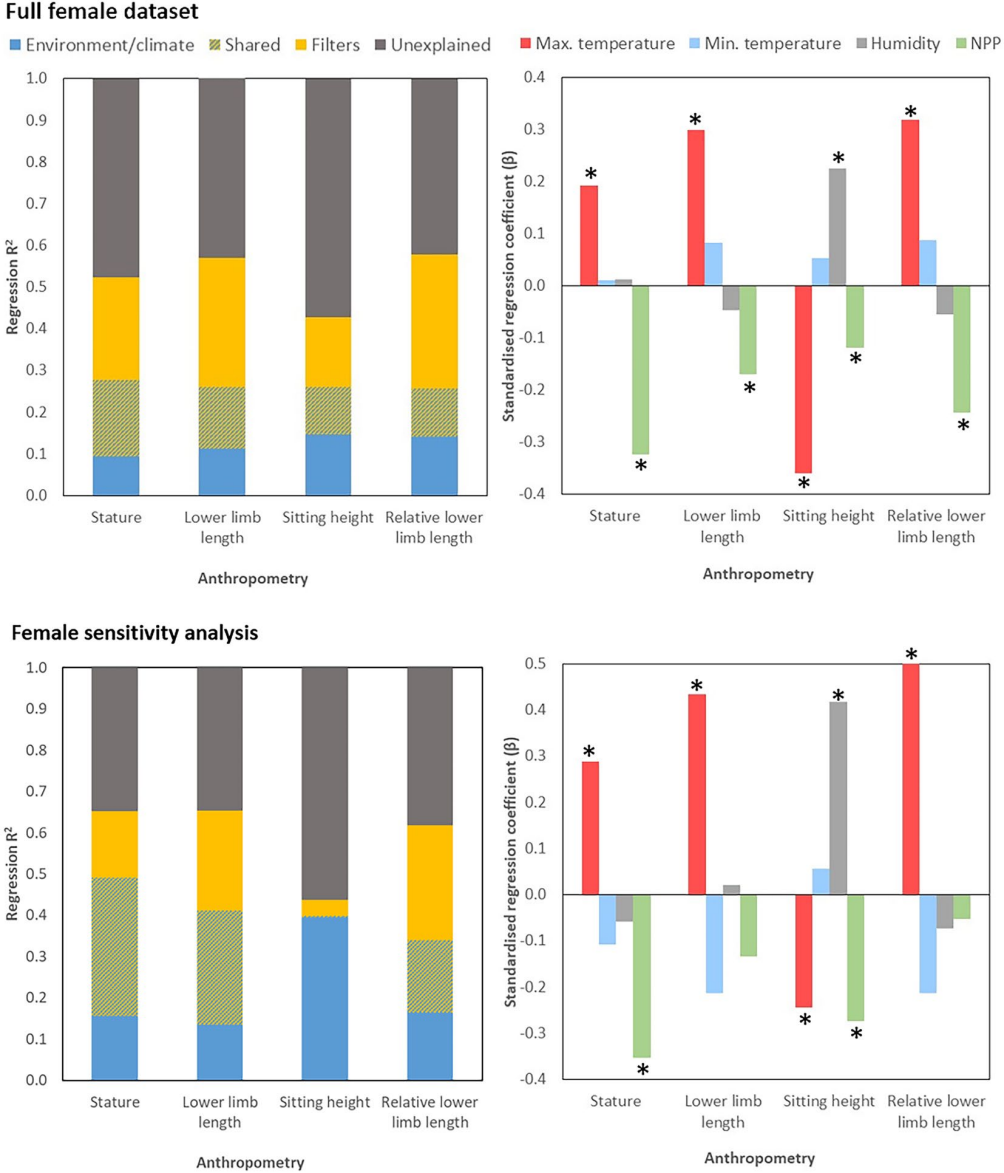
Results from the analysis of relationships between anthropometry and climatic/environmental variables for the female dataset (above: full; below; sensitivity analysis), adjusted for population history using spatial filters. Left: Variation explained by environment/climate, spatial filters, both together, or remaining unexplained by the models. Right: Standardised regression coefficients for environmental/climatic variables in explaining anthropometry, adjusting for population history. *NPP* net primary productivity. * Denotes p < 0.05 for variable in regression model, of which full details are in Supplementary Tables S7–S8. Above: full sample. Below: sensitivity analysis.

In the full female dataset, as with the male dataset, approximately half of the variation in each anthropometric variable is explained by the regression models (adjusted R^2^ = 0.41–0.56: Fig. 4, Table S7), with the least variance explained for sitting height and the most explained for absolute and relative lower limb lengths. The spatial filters uniquely explain more variation in anthropometry than the predictor variables in common with the male datasets. Maximum temperature is a positive predictor of all anthropometric variables, while minimum temperature does not feature significantly in any model. Temperature-adjusted humidity is a positive predictor of sitting height, and NPP is again a significant negative predictor of all anthropometry.

The female sensitivity analysis (Fig. 4, Table S8) demonstrates similar patterns despite greatly reduced sample size. Again a slightly higher proportion of the variance is explained than for the full dataset, and is lowest for sitting height and highest for relative LLL (adjusted R^2^ = 0.41 and 0.64 respectively: Table S8). Maximum temperature is a significant positive predictor of stature and absolute and relative LLLs and a negative predictor of sitting height, while minimum temperature does not feature significantly in any model. Humidity is a positive predictor of sitting height, and NPP is a significant negative predictor of stature and sitting height.

## Discussion

Stature, sitting height, and LLL (absolute and relative) show substantial patterning associated with population history, though more weakly for sitting height. In most models, the spatial filters account for a larger proportion of variation than the environmental/climatic variables, indicating that population history is equally, if not more important an influence on LLL, sitting height and stature than environmental and climatic variables. This finding is consistent with previous work on human skeletal variation^8,21,22,24,43–46,49^. As trunk length is less environmentally plastic than LLL^30–33^, we might predict a greater influence of population history than environmental/climatic variables on sitting height than LLL, but the opposite pattern is apparent and the underlying explanation remains unclear. Adjusting for population history, recent humans still conform to Allen’s “rule”, with absolutely and relatively longer lower limbs in hotter climates, while humidity and NPP also show significant associations with worldwide variation in stature, sitting height and LLL. Positive associations between temperature (or latitude as a proxy) and absolute or relative LLL have been reported previously^6–9^, while positive associations between stature and temperature probably reflect the effect of greater LLL as theoretically there is no clear advantage of increased stature per se for improved thermoregulation^11^, and sitting height is negatively associated with temperature in our analyses.

The pattern of results is largely consistent between the sexes, suggesting common influences of the climatic and environmental variables on morphology, as reported previously [e.g.,^6,9,11,39^]. The results were also similar between the full samples and pre-1950 sensitivity analyses, albeit with small variations, although it is notable that the proportion of variance explained by the models was greater in the sensitivity analyses than for their respective full datasets. This is consistent with previous work suggesting that ecogeographic patterns of variation in anthropometry have weakened in recent decades due to migration, changes in diet and behaviour, and increasing technological modification of climatic conditions in homes and workplaces^6^.

Temperature is a significant predictor in all models: maximum temperature is significant in 11 models, minimum temperature in 1 model, and both in 3 models. The standardised coefficients for temperature and NPP are generally the largest in absolute terms among the environmental and climatic variables. Maximum temperature is positively associated with stature and LLL (absolute and adjusted), except for stature in the male sensitivity analysis where no temperature variable featured. In contrast, maximum temperature is negatively associated with sitting height for all analyses except for the full male dataset, and minimum temperature is negatively associated with sitting height for the male but not the female analyses. While minimum temperatures have been shown to have the greatest effect on human skeletal morphology^13,19,20,43^, our results suggest that maximum temperatures play a more important role in influencing LLL, stature and sitting height, and support previous evidence from South American populations^9^ that for limb proportions, high and low temperatures play independent roles in influencing phenotype. However, the precise pattern of relationships between limb and trunk lengths/proportions differ between our work and that of Stinson^9^, whose analyses were restricted to correlations between variables in South American populations. Further work will be required to clarify whether this relates to differences in samples size and composition, study region, or other factors.

Although ecogeographic variation in sitting height has been less frequently studied, Stinson^9^ reported negative relationships between sitting height and temperature among South American populations. Trunk length appears to be less environmentally plastic than LLL (see above), and theoretical models show that body breadth is the key determinant of the surface area-volume ratio, while length (stature, but equally applicable to trunk length)^11,12,28^ and limb dimensions^28^ have little effect. Observed geographic variation in stature and pelvic breadth are argued to be consistent with this assumption^11,12^, so the negative association between trunk length and temperature is more difficult to explain. Heat stress may select for decreased metabolic heat production^26^ as well as strategies to dissipate heat, and reducing trunk length would decrease total mass, and so heat generation. Alternatively, in warm environments additional limb growth may be traded off against trunk growth to maximise surface area, although limb lengths may have minimal effect on surface area-volume ratio compared with body breadth^28^. As body breadth appears relatively developmentally constrained^44,52^, plasticity in limb and trunk lengths may be an important mechanism for shorter-term responses to climate.

Relative humidity is a consistent positive predictor of sitting height and a negative predictor of absolute and relative LLL in the male samples. Whether the sex difference in this pattern is meaningful or the result of sample sizes differences is unclear. The negative association between LLL and humidity is consistent with predictions that longer limbs do not aid thermoregulation in humid environments where evaporative heat loss is ineffective. Alternatively, higher parasite load in more humid environments may cause reduced growth, although the positive relationship between trunk length and humidity is less consistent with this explanation and the reason for a positive association between trunk length and relative humidity is unclear. One potential explanation is that to maintain critical visceral organ size while reducing body breadth, which shows a clear negative relationship to latitude/temperature in modern humans^11,12^, trunk length is increased. Further work will be required to test this scenario.

NPP is a significant negative predictor of all anthropometric data except LLL (absolute and relative) in the female sensitivity analysis. Rather than supporting the proposal that higher NPP provides greater resource availability and thus supports increased body size, these associations are consistent with the predictions of the “eNPP rule". Many populations in this study relied on agricultural subsistence, and high levels of seasonality in agricultural productivity in equatorial environments, often involving a hungry season where food is truly scarce, may impact long term growth [e.g.,^53–55^]. Alternatively, higher disease load in tropical regions^38^, which also have high NPP, results in a trade-off between growth and immune function, and may account for this association. A combination of seasonal food shortage and high disease load, given known interactions between nutrition and infection [e.g.,^53,55,56^], may ultimately explain the negative relationship between NPP and anthropometric data. As NPP is only a broad proxy for nutritional sufficiency and resource availability (see above), it would be desirable to find ways to more fully account for the potential effects of NPP and resource availability on global variation in human trunk and limb proportions.

In almost all analyses, NPP was more strongly associated with stature and sitting height than was temperature, while for relative and absolute LLL, the association was stronger with temperature than with NPP. This pattern may suggest that NPP/disease load have a stronger impact on total and sitting height than climate, while LLL shows a stronger influence of temperature. This is consistent with previous evidence that plastic responses in LLL to nutritional and/or disease stress are weaker than the impacts of long term climatic selection^57,58^.

In conclusion, this study indicates that population history plays as important a role in explaining variation in stature, sitting height and LLLs as climatic and environmental variables in a large global sample of recent humans. Nevertheless, even taking population history into account, our results still support Allen’s “rule”, which predicts longer limbs in hotter environments. Furthermore, we show that other eco-geographical correlates, particularly NPP, relate to stature and limb lengths, implying that resource availability and/or pathogen load also exert an important influence on these anthropometric characteristics. Proxies for population history generally explained a slightly greater proportion of the variance than environmental variables for LLL (absolute and relative) and stature, but not sitting height. NPP and temperature are generally the most consistent predictors of anthropometric variation: NPP is negatively associated with anthropometric variables and temperature is positively associated with LLL and stature, in accordance with Allen’s “rule”. Temperature is negatively associated with trunk length, for reasons which remain unclear. While the results show some interesting patterns, the study is unable to demonstrate causation and the mechanisms driving variation in stature and lower limb proportions remain uncertain. Nonetheless this study is novel in showing associations between anthropometry and both proxies for population history and resource availability, extending our understanding of the environmental factors potentially driving global variation in lower limb and trunk lengths, and stature.

## Methods

Data on stature, sitting height, and subischial LLL (stature minus sitting height) from adults of indigenous populations were collected from the literature (supplementary information Tables S1–S4, Fig. 1). Studies included had sampled ≥ 10 individuals from the general population (i.e. we excluded studies of specific medical conditions). If sitting height or LLL were not reported, values were calculated from raw individual data provided in the publication as the difference between mean stature and the available measurement (sitting height or  LLL), or using reported relative sitting height data. Only one measurement was permitted to be absent for inclusion in the dataset. Data were only included if it could be verified that trunk length was measured as sitting height, and LLL as subischial LLL as defined above. While the collection of data from the literature will inevitably introduce interobserver error, we expect this to be randomly distributed across the dataset and so to potentially weaken, but not bias, the reported patterns. There are well standardised protocols for measuring stature and sitting height and such pooling of data has previously been applied in other studies (e.g.^59^). The inclusion only of data where sitting height and subischial leg length are used to measure limb proportions maximised comparability across studies. Samples of fewer than 10 individuals, or where sample size was not given and there was no clear indication that sample size was large, were excluded from analyses, as were populations who were clearly recent migrants. Locations of study samples were taken from coordinates or maps and descriptions given in the publications and converted to decimal degrees. For the sensitivity analyses, date of measurement was recorded as when the data were collected according to the relevant publication, or where none was given/indicated, the year of publication. Since previous analyses have shown that climate-related patterns of phenotypic variation in humans have weakened in more recent populations, probably as a result of changes in diet, lifestyle and technology^6^, sensitivity analyses of data predating 1950 were also performed.

Temperature data came from BioClim variables derived from WorldClim dataset v 1.3^60^. Previous analyses suggest that temperature extremes drive climate-related phenotype [e.g.,^4,13,19–21,43^], so minimum temperature of the coldest month and maximum temperature of the warmest month were selected. Humidity data were from Jones and Wint^61^. As relative humidity is highly correlated with maximum temperature (Pearson correlation in our male dataset: r = − 0.85, p < 0.001), the standardised residual from the regression of mean maximum humidity on maximum temperature of the warmest month was used in analyses. NPP data were obtained from the Global Patterns in Net Primary Productivity dataset v1^62,63^. Climate and NPP variables were extracted for each sample location using DIVA-GIS 7.5 (diva-gis.org). While previous research has indicated a relationship between high altitude and relative lower limb proportions, much of this relationship is thought to be driven by nutritional and socioeconomic factors rather than high altitude hypoxia per se (e.g.^9,64,65^). Analyses initially included altitude, but strong covariation with other model variables (climate and NPP) meant that altitude was ultimately removed to ensure the models met relevant statistical assumptions. Given that altitude is thought to influence stature and limb proportions largely through nutrition and/or climate, altitude-related effects should still be accounted for in our models.

Given that most samples in the database are relatively recent (i.e., postdating 1870), we assume that recent climatic data are representative of the environmental conditions at the sample locations. Despite any changes over the last 150 years, modern data should adequately represent differences between populations sampled for this study. Such assumptions have been made for recent similar analyses of skeletal data^13,19–21,29,51,66,67^, and in such cases differences between coeval and modern environmental data are likely to have been greater on average than in our study.

To adjust for population history, we used a distance model taking into account dispersal patterns of humans following our evolution in Africa^51^. A pair-wise distance matrix was calculated in R v. 3.2.4^68^ following Betti et al.^51^, incorporating five waypoints to more realistically represent geographic dispersal routes from Africa^51^. This provides a framework to take into account the broad pattern of human dispersal, the detailed components of which are uncertain and subject to debate, while the calculation of the distance matrix incorporates the impact of spatial autocorrelation both within and outside of Africa. Given that current models suggest a wide geographical origin of our species within Africa^69^ and populations within the African continent demonstrate high levels of phenotypic, genetic, cultural and linguistic variation^70^, the methods used here incorporate spatial effects both within and outside the continent.

Stature, LLL, sitting height and relative LLL (standardised residual from a sex-specific ordinary least squares regression of LLL on sitting height) were analysed as dependent variables. To adjust for population history we used spatial eigenvector mapping (SEVM)^71,72^ in SAM v. 4.0^73^. Briefly, SEVM generates a set of eigenvectors or 'filters' using principal coordinates analysis to account for spatial autocorrelation. The software automatically selects the smallest number of filters to minimise Moran’s I (a measure of spatial autocorrelation) in the residuals in a partial correlation model that also includes the environmental/climatic variables^74^. The filters are then incorporated into an ordinary least squares regression to assess the separate and joint relationships of spatial and environmental variables with the response variables^71,72^. Truncation distance was set at 1500 km to optimise the spatial modelling over this distance as plots of Moran's I against distance indicated spatial autocorrelation was strongest at distances < 1500 km. Sexes were analysed separately, given known sexual dimorphism in body size and proportions, and the potential for differing patterns of selection and adaptation.

## Supplementary Information

Supplementary Information 1.

Supplementary Information 2.

## Data Availability

All data generated and analysed during this study are included in this published article and its Supplementary Information files.
