# Supplementary material for: Population history and ecology, in addition to climate, influence human stature and body proportions
Source: Sci Rep. 2021 Jan 11;11:274. doi: 10.1038/s41598-020-79501-w (PMC7801440; doi:10.1038/s41598-020-79501-w)
Supplement: Supplementary file 1 — Supplementary Information 1. [file 41598_2020_79501_MOESM1_ESM.pdf]

Population history and ecology, in addition to climate, influence human stature and body proportions

Emma Pomeroy[1], Jay T. Stock[2], and Jonathan C.K. Wells[3]

[1] Department of Archaeology, University of Cambridge, Downing Street, Cambridge CB2 3DZ, UK

[2] Department of Anthropology, Western University, London, Ontario, Canada, N6A 5C2

[3] UCL Great Ormond Street Institute of Child Health, University College London, Great Ormond Street, London WC1N 3JH, UK

Supplementary Information

Supplementary Tables

Table S1 and S2: Full datasets analysed in this study: see accompanying Excel file.

Table S3: Summary statistics for the anthropometric and environmental variables used in this study for the male sample (n = 571).

Variable	Mean	Standard deviation	Maximum	Minimum
Stature (cm)	165.3	5.7	182.1	144.4
Sitting height (cm)	85.5	3.4	95.2	75.9
Lower limb length (cm)	79.8	4.0	92.5	68.5
Date (years CE)	1944	39	2015	1870
Minimum temperature of the coldest month (°C)	5.4	13.1	24.6	-45.0
Maximum temperature of the warmest month (°C)	29.6	7.2	46.0	7.2
Net primary productivity (tonnes of carbon m^{-2} yr^{-1} x 10^{-6})	3.30	2.78	11.15	0.0
Temperature-adjusted relative humidity (%)	88.0	10.0	100.0	56.6

Table S4. Summary statistics for the anthropometric and environmental variables used in this study for the female sample (n = 268).

Variable	Mean	Standard deviation	Maximum	Minimum
Stature (cm)	153.8	5.7	166.5	136.0
Sitting height (cm)	80.2	3.4	87.8	70.4
Lower limb length (cm)	73.5	4.3	87.1	63.6
Date (years CE)	1959	42	2015	1885
Minimum temperature of the coldest month (°C)	4.2	15.2	23.8	-44.7
Maximum temperature of the warmest month (°C)	29.3	7.4	47.9	7.3
Net primary productivity (tonnes of carbon m^{-2} yr^{-1} x 10^{-6})	0.351	0.299	1.100	0.0
Mean maximum relative humidity (%)	66.9	15.0	96.8	36.5

Table S5. Regression models for stature and body proportions relative to environmental conditions for the full male sample (n = 571).

Dependent variable	R	R^2	Adjusted R^2	p	AIC	Partial regression R^2				Predictors	B	SE	Beta	p	Spatial filters (n)
						Predictors	Filters	Shared	Unexplained						
Stature	0.686	0.470	0.464	<0.001	3261	0.178	0.242	0.050	0.530	**Max. temperature**	**0.191**	**0.036**	**0.242**	**<0.001**	4
										Min. temperature	-0.012	0.022	-0.027	0.6	
										Humidity	-0.052	0.184	-0.009	0.8	
										**NPP**	**-7.431**	**0.770**	**-0.363**	**<0.001**	
Lower limb length	0.705	0.497	0.487	<0.001	2826	0.169	0.268	0.059	0.503	**Max. temperature**	**0.151**	**0.026**	**0.275**	**<0.001**	8
										**Min. temperature**	**0.050**	**0.016**	**0.164**	**0.002**	
										**Humidity**	**-0.771**	**0.131**	**-0.195**	**<0.001**	
										**NPP**	**-3.528**	**0.530**	**-0.248**	**<0.001**	
Sitting height	0.687	0.471	0.464	<0.001	2675	0.136	0.156	0.179	0.529	Max. temperature	0.003	0.021	0.006	0.9	5
										**Min. temperature**	**-0.046**	**0.013**	**-0.178**	**<0.001**	
										**Humidity**	**0.586**	**0.112**	**0.172**	**<0.001**	
										**NPP**	**-3.320**	**0.455**	**-0.272**	**<0.001**	
Relative lower limb length	0.706	0.498	0.487	<0.001	1258	0.190	0.252	0.056	0.502	**Max. temperature**	**0.041**	**0.007**	**0.298**	**<0.001**	9
										**Min. temperature**	**0.015**	**0.004**	**0.191**	**<0.001**	
										**Humidity**	**-0.242**	**0.033**	**-0.241**	**<0.001**	
										**NPP**	**-0.703**	**0.134**	**-0.195**	**<0.001**	

Relative lower limb length = standardised residual from regression of lower limb length on sitting height. Max. temperature = maximum temperature of the warmest month; Min. temperature = Minimum temperature of the coldest month; Humidity = standardised residual of maximum relative humidity adjusted for maximum temperature of the warmest month; NPP = Net Primary Productivity. Statistically significant predictors of the dependent variable at $p \leq 0.05$ are shown in bold.

Table S6. Regression models for stature and body proportions relative to environmental conditions for the pre-1950 male sample (sensitivity analysis: n = 323).

Dependent variable	R	R^2	Adjusted R^2	p	AIC	Partial regression R^2				Predictors	B	SE	Beta	p	Spatial filters (n)
						Predictors	Filters	Shared	Unexplained						
Stature	0.777	0.604	0.591	<0.001	1741	0.039	0.319	0.246	0.396	Max. temperature	0.073	0.047	0.091	0.1	7
										Min. temperature	-0.029	0.027	-0.067	0.3	
										Humidity	-0.316	0.214	-0.058	0.1	
										**NPP**	**-3.953**	**1.049**	**-0.182**	**<0.001**	
Lower limb length	0.790	0.624	0.611	<0.001	1503	0.116	0.363	0.145	0.376	**Max. temperature**	**0.152**	**0.034**	**0.266**	**<0.001**	8
										Min. temperature	-0.001	0.019	-0.004	0.9	
										**Humidity**	**-0.922**	**0.145**	**-0.238**	**<0.001**	
										**NPP**	**-1.950**	**0.727**	**-0.127**	**0.008**	
Sitting height	0.733	0.537	0.527	<0.001	1441	0.116	0.140	0.281	0.463	**Max. temperature**	**-0.078**	**0.029**	**-0.165**	**0.007**	4
										**Min. temperature**	**-0.040**	**0.017**	**-0.158**	**0.017**	
										**Humidity**	**0.413**	**0.135**	**0.128**	**0.002**	
										**NPP**	**-2.428**	**0.634**	**-0.191**	**0.002**	
Relative lower limb length	0.787	0.619	0.607	<0.001	619	0.145	0.344	0.130	0.381	**Max. temperature**	**0.038**	**0.008**	**0.264**	**<0.001**	7
										Min. temperature	0.004	0.005	0.046	0.4	
										**Humidity**	**-0.253**	**0.037**	**-0.258**	**<0.001**	
										**NPP**	**-0.409**	**0.185**	**-0.105**	**0.028**	

Relative lower limb length = standardised residual from regression of lower limb length on sitting height. Max. temperature = maximum temperature of the warmest month; Min. temperature = Minimum temperature of the coldest month; Humidity = standardised residual of maximum relative humidity adjusted for maximum temperature of the warmest month; NPP = Net Primary Productivity. Statistically significant predictors of the dependent variable at $p \leq 0.05$ are shown in bold.

Table S7. Regression models for stature and body proportions relative to environmental conditions for the full female sample (n = 268).

Dependent variable	R	R^2	Adjusted R^2	p	AIC	Partial regression R^2				Predictors	B	SE	Beta	p	Spatial filters (n)
						Predictors	Filters	Shared	Unexplained						
Stature	0.724	0.524	0.510	<0.001	1513	0.093	0.247	0.183	0.476	**Max. temperature**	**0.147**	**0.048**	**0.193**	**0.003**	5
										Min. temperature	0.004	0.026	0.010	0.9	
										Humidity	0.072	0.322	0.013	0.8	
										**NPP**	**-6.130**	**1.001**	**-0.323**	**<0.001**	
Lower limb length	0.754	0.569	0.554	<0.001	1342	0.112	0.309	0.149	0.431	**Max. temperature**	**0.173**	**0.034**	**0.299**	**<0.001**	6
										Min. temperature	0.023	0.019	0.082	0.2	
										Humidity	-0.196	0.236	-0.046	0.4	
										**NPP**	**-2.442**	**0.715**	**-0.170**	**<0.001**	
Sitting height	0.653	0.427	0.405	<0.001	1301	0.146	0.166	0.115	0.573	**Max. temperature**	**-0.166**	**0.032**	**-0.359**	**<0.001**	7
										Min. temperature	0.012	0.017	0.053	0.5	
										**Humidity**	**0.776**	**0.217**	**0.225**	**<0.001**	
										**NPP**	**-1.369**	**0.706**	**-0.119**	**0.05**	
Relative lower limb length	0.761	0.579	0.564	<0.001	552	0.141	0.322	0.116	0.421	**Max. temperature**	**0.043**	**0.008**	**0.319**	**<0.001**	6
										Min. temperature	0.006	0.004	0.087	0.2	
										Humidity	-0.055	0.054	-0.055	0.3	
										**NPP**	**-0.815**	**0.172**	**-0.244**	**<0.001**	

Relative lower limb length = standardised residual from regression of lower limb length on sitting height. Max. temperature = maximum temperature of the warmest month; Min. temperature = Minimum temperature of the coldest month; Humidity = standardised residual of maximum relative humidity adjusted for maximum temperature of the warmest month; NPP = Net Primary Productivity. Statistically significant predictors of the dependent variable at p ≤ 0.05 are shown in bold.

Table S8. Regression models for stature and body proportions relative to environmental conditions for the pre-1950 female sample (sensitivity analysis: n = 94).

Dependent variable	R	R^2	Adjusted R^2	p	AIC	Partial regression R^2				Predictors	B	SE	Beta	p	Spatial filters (n)
						Predictors	Filters	Shared	Unexplained						
Stature	0.808	0.652	0.628	<0.001	523	0.155	0.161	0.336	0.348	**Max. temperature**	**0.284**	**0.084**	**0.288**	**<0.001**	3
										Min. temperature	-0.045	0.046	-0.109	0.3	
										Humidity	-0.309	0.575	-0.059	0.6	
										**NPP**	**-7.547**	**1.798**	**-0.354**	**<0.001**	
Lower limb length	0.809	0.654	0.635	<0.001	462	0.135	0.242	0.277	0.346	**Max. temperature**	**0.317**	**0.061**	**0.433**	**<0.001**	2
										Min. temperature	-0.065	0.034	-0.213	0.06	
										Humidity	0.081	0.414	0.021	0.8	
										NPP	-2.127	1.242	-0.134	0.09	
Sitting height	0.661	0.437	0.412	<0.001	427	0.395	0.038	0.004	0.563	**Max. temperature**	**-0.117**	**0.05**	**-0.244**	**0.021**	1
										Min. temperature	0.011	0.027	0.056	0.7	
										**Humidity**	**1.073**	**0.248**	**0.418**	**<0.001**	
										**NPP**	**-2.841**	**1.006**	**-0.274**	**0.006**	
Relative lower limb length	0.786	0.618	0.596	<0.001	192	0.164	0.279	0.175	0.382	**Max. temperature**	**0.082**	**0.014**	**0.500**	**<0.001**	2
										Min. temperature	-0.015	0.008	-0.213	0.07	
										Humidity	-0.065	0.097	-0.074	0.5	
										NPP	-0.184	0.292	-0.052	0.5	

Relative lower limb length = standardised residual from regression of lower limb length on sitting height. Max. temperature = maximum temperature of the warmest month; Min. temperature = Minimum temperature of the coldest month; Humidity = standardised residual of maximum relative humidity adjusted for maximum temperature of the warmest month; NPP = Net Primary Productivity. Statistically significant predictors of the dependent variable at $p \leq 0.05$ are shown in bold.
